# Clinical Desire for an Artificial Intelligence–Based Surgical Assistant System: Electronic Survey–Based Study

**DOI:** 10.2196/17647

**Published:** 2020-05-15

**Authors:** Soo Jin Park, Eun Ji Lee, Se Ik Kim, Seong-Ho Kong, Chang Wook Jeong, Hee Seung Kim

**Affiliations:** 1 Department of Obstetrics and Gynecology Seoul National University College of Medicine Seoul Republic of Korea; 2 Department of Surgery Seoul National University College of Medicine Seoul Republic of Korea; 3 Department of Urology Seoul National University College of Medicine Seoul Republic of Korea

**Keywords:** artificial intelligence, solo surgery, laparoscopic surgery

## Abstract

**Background:**

Techniques utilizing artificial intelligence (AI) are rapidly growing in medical research and development, especially in the operating room. However, the application of AI in the operating room has been limited to small tasks or software, such as clinical decision systems. It still largely depends on human resources and technology involving the surgeons’ hands. Therefore, we conceptualized AI-based solo surgery (AISS) defined as laparoscopic surgery conducted by only one surgeon with support from an AI-based surgical assistant system, and we performed an electronic survey on the clinical desire for such a system.

**Objective:**

This study aimed to evaluate the experiences of surgeons who have performed laparoscopic surgery, the limitations of conventional laparoscopic surgical systems, and the desire for an AI-based surgical assistant system for AISS.

**Methods:**

We performed an online survey for gynecologists, urologists, and general surgeons from June to August 2017. The questionnaire consisted of six items about experience, two about limitations, and five about the clinical desire for an AI-based surgical assistant system for AISS.

**Results:**

A total of 508 surgeons who have performed laparoscopic surgery responded to the survey. Most of the surgeons needed two or more assistants during laparoscopic surgery, and the rate was higher among gynecologists (251/278, 90.3%) than among general surgeons (123/173, 71.1%) and urologists (35/57, 61.4%). The majority of responders answered that the skillfulness of surgical assistants was “very important” or “important.” The most uncomfortable aspect of laparoscopic surgery was unskilled movement of the camera (431/508, 84.8%) and instruments (303/508, 59.6%). About 40% (199/508, 39.1%) of responders answered that the AI-based surgical assistant system could substitute 41%-60% of the current workforce, and 83.3% (423/508) showed willingness to buy the system. Furthermore, the most reasonable price was US $30,000-50,000.

**Conclusions:**

Surgeons who perform laparoscopic surgery may feel discomfort with the conventional laparoscopic surgical system in terms of assistant skillfulness, and they may think that the skillfulness of surgical assistants is essential. They desire to alleviate present inconveniences with the conventional laparoscopic surgical system and to perform a safe and comfortable operation by using an AI-based surgical assistant system for AISS.

## Introduction

Artificial intelligence (AI) has been rapidly developing in recent years, and relevant research is being actively conducted in the health care field through deep learning and big data technology [1]. AI applied in the medical area can be divided into the following two categories: virtual and physical AI. Virtual AI includes the programs that can help clinical diagnosis, whereas physical AI involves smart operating rooms, nanorobots, and patient-assistance systems [2]. In particular, physical AI in the operating room can assist the operator or replace the assistant during surgery [2,3]. For instance, the da Vinci surgical system, which is the first computer-based robotic surgical system approved by the US Food and Drug Administration in 2000, has been widely used for minimally invasive surgery, including laparoscopic surgery. The demand for the robotic surgical system is rapidly increasing in the surgical areas of gynecology, general surgery, and urology [4]. This increase in demand is due to reduced surgeon fatigue and improved surgical access through ergonomic instruments and three-dimensional imaging [4,5].

However, the current robotic surgical system still depends on coordination of the human eye and hand, which is insufficient in terms of autonomy or interaction [6,7]. In particular, the injection of carbon dioxide and insertion of trocars into the peritoneal cavity are still performed by surgeons without the aid of a robotic surgical system, and the laparoscopic camera and instruments are adjusted manually to the target by surgeons. Thus, an automated robotic surgical system that is better than the current master-slave approach may be expected to reduce human error and thereby improve the quality of surgery. Up to now, relevant studies have mainly focused on the development of robots capable of performing short surgical tasks, such as knot tie and needle insertion [8,9], and the application of voice interaction technology during surgery may be one of the crucial elements that should be developed in an AI-based surgical assistant system [10-12].

Nevertheless, high medical cost may be one of the barriers to the adoption of an AI-based surgical assistant system [13], and it is not yet know how this system will improve the quality of surgery or reduce human resources effectively. Therefore, we conceptualized AI-based solo surgery (AISS) that was defined as laparoscopic surgery conducted by only one surgeon with support from an AI-based surgical assistant system and considered the clinical desire for AISS via an electronic survey (e-survey).

An e-survey has been a common method of research in human and social sciences since the 1990s. In the case of research using a web-based questionnaire, it is possible to attach pictures or materials in order to avoid response omission as much as possible and avoid inconsistent or out-of-frame results. Besides, data can be effectively organized and archived without paper resources, and distribution via email can be quickly done through a URL [14]. Moreover, by distributing the web questionnaire via email, it is possible to limit the target respondents to people belonging to a specific community so that the questionnaire survey is conducted for experts in the relevant field.

Therefore, we performed an e-survey to investigate the clinical desire for an AI-based surgical assistant system for AISS as compared with the current laparoscopic surgical system and to determine the reasonable cost of such an AI-based surgical assistant system for AISS.

## Methods

### Survey

We surveyed gynecologists from the Korean Society of Obstetrics and Gynecology, urologists from the Korean Urologic Association, and general surgeons from the Korean Surgical Society between June and August 2017 through nownsurvey (ELIMNET Co, Ltd) [15], a commercially available e-survey platform. In this survey, the AI-based surgical assistant system for AISS was considered to have the following functions: camera automatic recognition and operation function through voice commands; action as an assistant by manipulating surgical instruments through automatic screen recognition and voice commands; and smart storage for recognizing, indexing, and storing surgical procedures while recording specific events. There were a total of 13 questions that included six items about the responder’s experience, two about limitations of the conventional laparoscopic surgical system, and five about the clinical desire for an AI-based surgical assistant system for AISS (Table 1). We estimated that 5000 gynecologists, 7000 general surgeons, and 2500 urologists would participate in the survey. This study was approved by the Institutional Review Board of Seoul National University Hospital (approval no: 1910-131-1072).

**Table 1:** Questionnaire details.

Variable and question number		Question	
**Experience**			
	1		What type of hospital do you work at?
	2		What department do you work in?
	3		How many patients do you perform laparoscopic surgery in monthly?
	4		How many assistants do you need during laparoscopic surgery?
	5		What kinds of assistants do you want during laparoscopic surgery?
	6		How important is the skillfulness of your assistant for successful laparoscopic surgery?
**Limitation**			
	7		What are your discomforts during laparoscopic surgery owing to inexperienced camera assistants? (multiple choice)
	8		What are your discomforts during laparoscopic surgery owing to inexperienced laparoscopic instrument assistants? (multiple choice)
**Desire**			
	9		What functions do you expect to be included in the AI^a^-based surgical assistant system for AISS^b^? (multiple choice)
	10		What percentage of your assistant’s function will the AI-based surgical assistant system for AISS replace?
	11		Would you want to buy the AI-based surgical assistant system for AISS if it thrives?
	12		Why would you want to buy the AI-based surgical assistant system for AISS? (multiple choice)
	13		How much would you like to pay for the AI-based surgical assistant system for AISS?

^a^AI: artificial intelligence.

^b^AISS: artificial intelligence–based solo surgery.

### Data Analysis

We analyzed each question by using descriptive statistics. Additionally, we analyzed all the respondents, and the response rate was 3.5%. Each item in the questionnaire was stratified according to the surgeons’ fields as follows: gynecologists, urologists, and general surgeons. Categorical variables were analyzed with the chi-square test or Fisher exact test using the statistical software SPSS 20.0 (IBM Corp, Armonk, New York, USA). A *P* value <.05 was considered statistically significant.

## Results

### Experience

Table 2 shows the demographic data of the responders. A total of 508 people responded to the questionnaire, and there were 278 gynecologists, 173 general surgeons, and 57 urologists. Among the three surgeon fields, most of the urologists (49/57, 86.0%) worked at a university hospital, whereas relatively many gynecologists (67/278, 24.1%) worked as general practitioners. Moreover, most of the urologists (43/57, 75.4%) performed laparoscopic surgery in less than 10 cases per month, whereas relatively many general surgeons performed laparoscopic surgery in 31 or more cases per month (40/173, 23.1%). In terms of the number of assistants during laparoscopic surgery, 38.6% (22/57) of urologists required one or less assistant, whereas 90.3% (251/278) of gynecologists required two or more assistants.

**Figure 1:**
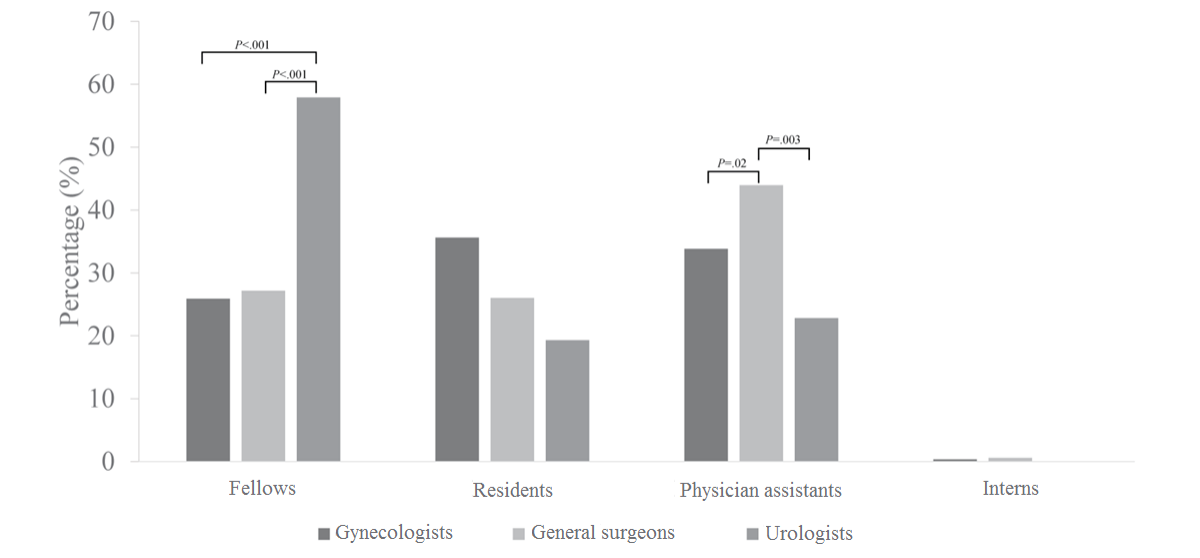
Comparison of assistants preferred during laparoscopic surgery.

**Figure 2:**
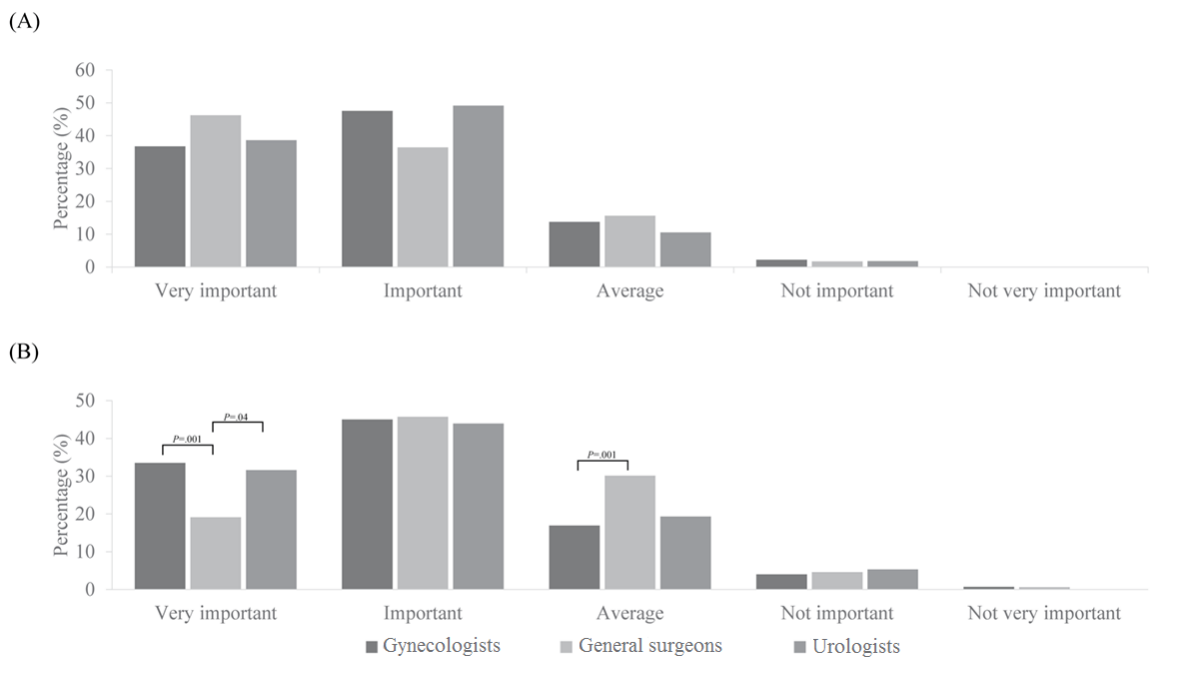
Comparison of the importance of the skillfulness of (A) camera and (B) instrument assistants.

In terms of the preferred assistant during laparoscopic surgery, most of the urologists (33/57, 57.9%) preferred fellows, whereas many general surgeons (76/173, 43.9%) preferred physician assistants (Figure 1). With regard to the importance of the skillfulness of assistants, who manipulate cameras or instruments, for successful laparoscopic surgery, most of the responders indicated “very important” or “important,” regardless of the surgeon field. Although the trend was similar among the three surgeon fields with regard to the camera assistant, general surgeons (33/173, 19.1%) relatively underestimated the importance of the skillfulness of instrument assistants as compared with gynecologists (93/278, 33.5%) or urologists (18/57, 31.6%) (Figure 2).

**Table 2:** Demographic data.

Answers		Total (N=508), n (%)	Gynecologists (N=278), n (%)	General surgeons (N=173), n (%)	Urologists (N=57), n (%)	*P* value
**Working hospital**						**<.001**
	University hospital	306 (60.2)	146 (52.5)	111 (64.2)	49 (86.0)	
	General hospital	76 (15.0)	34 (12.2)	36 (20.8)	6 (10.5)	
	Semi hospital	49 (9.6)	31 (11.2)	17 (9.8)	1 (1.8)	
	General practitioner	77 (15.2)	67 (24.1)	9 (5.2)	1 (1.8)	
**Total number of laparoscopic surgeries per month**						**<.001**
	0-10	232 (45.7)	136 (48.9)	53 (30.6)	43 (75.4)	
	11-30	181 (35.6)	89 (32.0)	80 (46.2)	12 (21.1)	
	≥31	95 (18.7)	53 (19.1)	40 (23.1)	2 (3.5)	
**Number of assistants during laparoscopic surgery**						**<.001**
	0	1 (0.2)	0 (0.0)	1 (0.6)	0 (0.0)	
	1	98 (19.3)	27 (9.7)	49 (28.3)	22 (38.6)	
	2	349 (68.7)	214 (77.0)	106 (61.3)	29 (50.9)	
	≥3	60 (11.8)	37 (13.3)	17 (9.8)	6 (10.5)	

### Limitation

Table 3 shows the responses to questions on the surgeons’ discomforts related to inexperienced camera and instrument assistants for the conventional laparoscopic surgical system. With regard to the camera assistant, 84.8% (431/508) of the responders were unsatisfied with unskilled movement of the camera in the intended direction. In particular, gynecologists (69/278, 24.8%) had more complaints about contamination of the camera lens by blood or body fluid as compared with general surgeons (26/173, 15.0%) or urologists (6/57, 10.5%). With regard to the instrument assistant, 59.6% (303/508) of the responders were unsatisfied with unskilled movement of the instruments in the intended direction. In particular, general surgeons (103/173, 59.5%) had more complaints about tissue damage or bleeding by inappropriate traction and urologists (24/57, 42.1%) had more complaints about collision between the instruments as compared with the other surgeons. On the other hand, gynecologists (32/278, 11.5%) had more complaints about swaying of the instruments as compared with the other surgeons.

**Table 3:** Surgeons’ discomforts regarding the conventional laparoscopic surgical system.

Discomfort		Total (N=508), n (%)	Gynecologists (N=278), n (%)	General surgeons (N=173), n (%)	Urologists (N=57), n (%)	*P* value
**Camera assistant**						
	Unskilled movement of the camera in the intended direction	431 (84.8)	227 (81.7)	151 (87.3)	53 (93.0)	.05
	Dizziness due to excessive camera movement	174 (34.3)	96 (34.5)	61 (35.3)	17 (29.8)	.75
	Inappropriate field of view due to excessive zoom in or out	156 (30.7)	80 (28.8)	59 (34.1)	17 (29.8)	.49
	Condensation on the camera lens	123 (24.2)	76 (27.3)	39 (22.5)	8 (14.0)	.08
	Contamination of the camera lens by blood or body fluid	101 (19.9)	69 (24.8)	26 (15.0)	6 (10.5)	.01
	Blurriness of the camera	101 (19.9)	57 (20.5)	32 (18.5)	12 (21.1)	.85
**Instrument assistant**						
	Unskilled movement of the instruments in the intended direction	303 (59.6)	172 (61.9)	94 (54.3)	37 (64.9)	0.20
	Inappropriate tissue traction due to lack of power to pull or push	196 (38.6)	102 (36.7)	79 (45.7)	15 (26.3)	0.02
	Dangerous movement of the instruments outside the camera view	145 (28.5)	75 (27.0)	47 (27.2)	23 (40.4)	0.11
	Tissue damage or bleeding by inappropriate traction	189 (37.2)	63 (22.7)	103 (59.5)	23 (40.4)	<.001
	Collision between the instruments	126 (24.8)	57 (20.5)	45 (26.0)	24 (42.1)	.002
	Insufficient removal of intra-abdominal smoke during surgery	77 (15.2)	52 (18.7)	18 (10.4)	7 (12.3)	.047
	Swaying of the instruments	44 (8.7)	32 (11.5)	9 (5.2)	3 (5.3)	.04

### Desire

**Figure 3:**
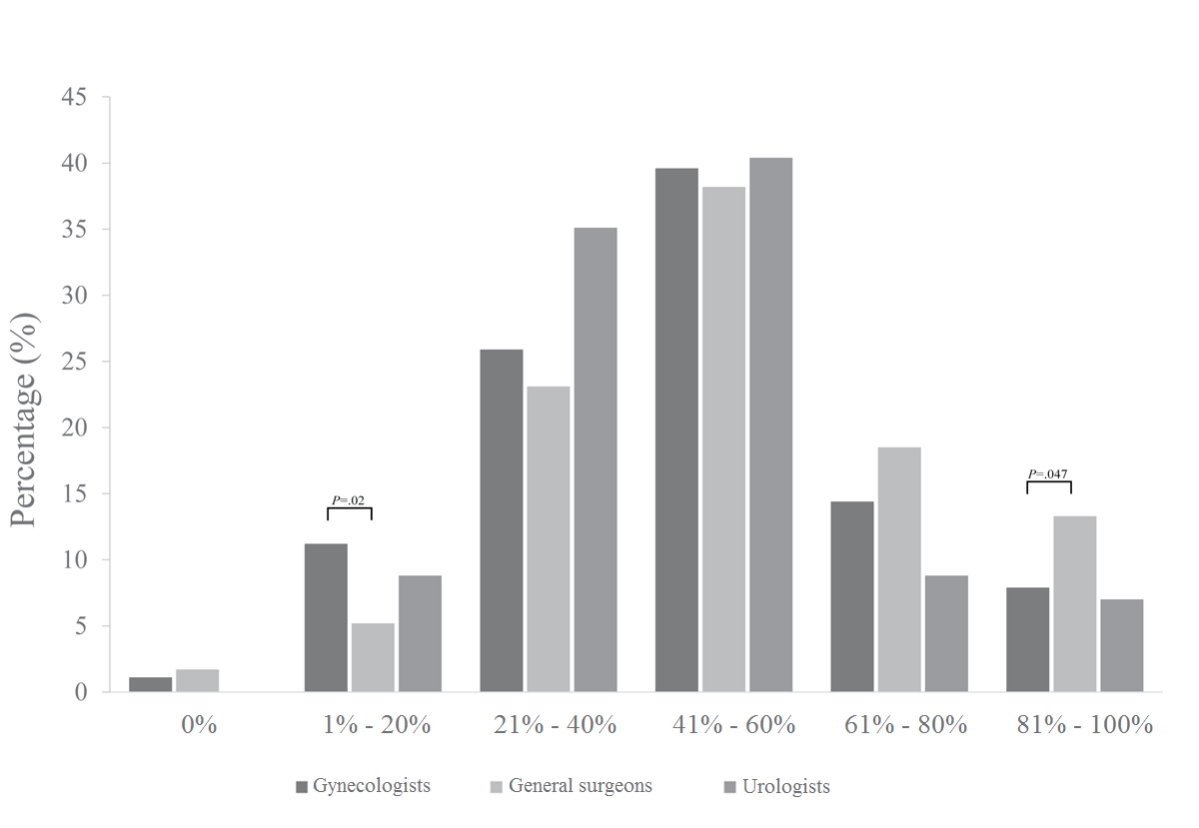
Expectations about how much an artificial intelligence–based surgical assistant system can replace the existing workforce.

Table 4 depicts the functions that should be included in an AI-based surgical assistant system for AISS to overcome the limitations of the current laparoscopic surgical system. More than half of the responders preferred intuitive and easy maneuverability (308/508, 60.6%), a demister and self-cleaning system for the laparoscopic camera lens (326/508, 64.2%), and safety for minimizing tissue damage (279/508, 54.9%). In particular, more urologists (29/57, 50.9%) desired fast running by minimizing time delay as compared with gynecologists (86/278, 30.9%) and general surgeons (67/173, 38.7%). However, interest in the autosave or voice command system for special events during the operation was the lowest among the three surgeon fields. In terms of the possibility that the AI-based surgical assistant system for AISS can replace the functions of assistants, about 40% (199/508, 39.1%) of responders expected it to substitute 41%-60% of the existing workforce (Figure 3).

When asked about the purchase intention and reasonable price to buy the AI-based surgical assistant system for AISS, 83.3% (423/508) of all responders wanted to buy the system. The most common reason for wanting to buy the system was the comfort of laparoscopic surgery (257/508, 50.6%). In particular, general surgeons had a relatively strong desire to decrease the burden of repetitive training for assistants, whereas they had less interest in the reduction of the operation time by purchasing the AI-based surgical assistant system for AISS as compared with gynecologists. Regarding the reasonable price for the system, 29.7% (151/508) of the responders had a willingness to pay US $30,000-50,000 (Table 5).

**Table 4:** Functions that should be included in an artificial intelligence–based surgical assistant system.

Function	Total (N=508), n (%)	Gynecologists (N=278), n (%)	General surgeons (N=173), n (%)	Urologists (N=57), n (%)	*P* value
Intuitive and easy maneuverability	308 (60.6)	164 (59.0)	108 (62.4)	36 (63.2)	.71
Demister and self-cleaning system for the laparoscopic camera lens	326 (64.2)	186 (66.9)	108 (62.4)	32 (56.1)	.26
Safety for minimizing tissue damage	279 (54.9)	150 (54.0)	97 (56.1)	32 (56.1)	.89
Reasonable size of the instruments avoiding operator disturbance	248 (48.8)	130 (46.8)	86 (49.7)	32 (56.1)	.42
Stabilization of the laparoscopic camera and instruments	220 (43.3)	122 (43.9)	69 (39.9)	29 (50.9)	.33
Stable movements not causing dizziness	208 (40.9)	114 (41.0)	65 (37.6)	29 (50.9)	.21
Functions for complex movements, such as axial rotation of the 30-degree camera and manipulation of the flexible scope	213 (41.9)	107 (38.5)	80 (46.2)	26 (45.6)	.22
Fast running by minimizing time delay	182 (35.8)	86 (30.9)	67 (38.7)	29 (50.9)	.01
Autosave or voice command system for special events during the operation	139 (27.4)	76 (27.3)	46 (26.6)	17 (29.8)	.89

**Table 5:** Purchase intention and reasonable price to buy the artificial intelligence–based surgical assistant system.

Answers			Total (N=508), n (%)		Gynecologists (N=278), n (%)		General surgeons (N=173), n (%)		Urologists (N=57), n (%)		*P* value
Purchase intention			423 (83.3)		222 (79.9)		151 (87.3)		50 (87.7)		.08
**Reason to buy the system**											
	Comfort of laparoscopic surgery	257 (50.6)		142 (64.0)		84 (55.6)		31 (62.0)		.27	
	Improved safety and maturity of laparoscopic surgery	245 (48.2)		126 (56.8)		85 (56.3)		34 (68.0)		.31	
	Decreased number of assistants	204 (40.2)		101 (45.5)		75 (49.7)		28 (56.0)		.37	
	Decreased burden of repetitive training for assistants	197 (38.8)		95 (42.8)		82 (54.3)		20 (4.0)		.06	
	Reduced operation time	119 (23.4)		74 (33.3)		27 (17.9)		18 (36.0)		.002	
	Improved convenience of research based on the autosave function	114 (22.4)		59 (26.6)		39 (25.8)		16 (32.0)		.68	
**Reasonable price (US$)**											**.04**
	<30,000	87 (17.1)		61 (21.9)		21 (12.1)		5 (8.8)			
	30,000-50,000	151 (29.7)		83 (29.9)		53 (30.6)		15 (26.3)			
	50,000-100,000	139 (27.4)		77 (27.7)		43 (24.9)		19 (33.3)			
	100,000-150,000	79 (15.6)		33 (11.9)		36 (20.8)		10 (17.5)			
	150,000-200,000	28 (5.5)		11 (4.0)		13 (7.5)		4 (7.0)			
	≥200,000	24 (4.7)		13 (4.7)		7 (4.0)		4 (7.0)			

## Discussion

### Principal Findings

This study involved a survey about the clinical desire for an AI-based surgical assistant system for AISS among surgeons who currently perform laparoscopic surgery. In this survey, we identified the importance of assistants and the discomforts with the conventional laparoscopic surgical system and determined surgeons’ expectations and demands for new AI-based robotic surgery aids.

### Experience

In terms of experience, gynecologists were more likely to have two assistants than general surgeons and urologists. The reason is that gynecologists may use a uterine manipulator frequently during laparoscopic gynecologic surgery [16]. Therefore, gynecologists commonly require two or more assistants for laparoscopic surgery, including two assistants who hold a laparoscopic camera and a uterine manipulator.

On the other hand, urologists’ preference for fellows as surgical assistants could be related to more common practice in university hospitals. Moreover, urologists can be less dependent on residents during surgery, which may be similar for general surgeons who prefer physician assistants as surgical assistants. Furthermore, most of the responders valued the skillfulness of surgical assistants who manipulate the laparoscopic camera and instrument assistants, because the extent of assistant experience may be closely related to the operation time and complication rate [17]. Recently, in the Republic of Korea, owing to the implementation of the special act regarding an 80-hour workweek for residents, their working time has reduced, and thereby, the number of cases of surgical training has reduced [18]. In contrast, physician assistants are still useful for coordination in the operating room because of their high level of proficiency based on repetitive work [19]. Therefore, most surgeons seem to prefer fellows or physician assistants who are proficient in laparoscopic surgery rather than residents or interns because of their skillfulness as surgical assistants in the Republic of Korea.

### Limitation

In terms of limitation, most of the surgeons felt uncomfortable with camera assistants when they showed unskilled movement of the camera in the intended direction and instrument assistants when they showed unskilled movement of the instruments in the intended direction. This result is consistent with the finding that most of the surgeons considered the skillfulness of surgical assistants as “very important” or “important,” regardless of the field.

### Desire

In terms of desire, the essential functions desired to be present in an AI-based surgical assistant system for AISS were intuitive and easy maneuverability, a demister and self-cleaning system for the laparoscopic camera lens, and safety for minimizing tissue damage. Interestingly, only 10%-20% of surgeons complained about discomfort regarding the camera lens or foreign objects, whereas a high percentage of surgeons desired a self-cleaning system for AISS. These findings seem to be associated with the role of surgical assistants in camera cleaning when using the conventional laparoscopic surgical system, which is perceived as an essential function by the operator, and the absence of an uncomfortable feeling with the current system.

Notably, more than 80% of the responders intended to buy the AI-based surgical assistant system for AISS, and the reasons for buying it were comfort of laparoscopic surgery and improved safety and maturity of laparoscopic surgery. Considering the results from the questions on the conventional laparoscopic surgical system, surgeons showed a tendency to overcome current constraints regarding laparoscopic surgery with the AI-based surgical assistant system, especially with regard to the skillfulness of assistants.

The majority of responders anticipated that the introduction of the AI-based surgical assistant system would replace the existing workforce by 41%-60%. Therefore, an AI-based surgical assistant system for AISS could be a great solution in university hospitals where resident working hours are regulated (eg, 80-hour resident special act in Korea and The European Working Time Directive in Europe) [18,20]. Of course, there may be some opinions concerning undertraining of residents, but the introduction of educational tools, such as simulation training systems, is a possible alternative [17,21].

### Issues Related to Practical Application

Before adopting and introducing an AI-based surgical assistant system in the surgical field, ethical and legal responsibilities should be discussed through consensus of medical, legal, and administrative experts and others. Additionally, although not included in this survey, the recent development of AI is likely to include explainable AI, a concept contrasted with previous black-box AI, in the development of new technologies.

At the time of the introduction of robotic surgery, which is being actively used presently, many experts had discussed ethical issues [22-24]. Current robotic surgery is a master-slave system, with the surgeon having most of the responsibility, making it easy to discuss ethical issues. However, in the case of an autonomous AI-based surgical assistant system, there may be controversy regarding the responsibility for harm and injuries caused to the patient during the robotic surgery, and social discussions about this need to be carried out for the adoption of an AI-based surgical assistant system [24,25].

Explainability should be considered when newly developing AI-based surgical assistant systems. Current AI-based medical programs involving deep learning and machine learning techniques lack explainability, hindering the dependence of medical professionals on conclusions from these programs. Therefore, considering the characteristics of surgical procedures that are repeated continuously with small and large decisions, it is expected that explainability will be essential for the interaction between the machine and the operator and should be incorporated in the development of AI-based robotic assistance systems that contribute to these procedures [26].

### Strengths and Weaknesses

This report is based on a survey among experts who have been actively performing laparoscopic surgery in various fields. To the best of our knowledge, this is the first report showing the clinical need for an AI-based surgical assistant system for AISS according to an e-survey. Moreover, this study is meaningful because we could identify the unmet need of clinicians for an AI-based system for AISS, which could be developed soon. However, this study has some limitations. First, it was challenging to check the exact response rate through the mailing system used in this study, which could act as a bias, and thus, the results of this study should be interpreted carefully. However, we could assume that the questionnaire was answered by our targeted responders because most of the responders mentioned that they performed more than one surgery per month. Second, the specific national health insurance system controlled by the government in the Republic of Korea could affect the expected value of an AI-based surgical assistant system for AISS, and the finding should be complemented by international surveys later. Third, the validity and reliability of the items in the questionnaire could not be confirmed because there has been no previous comparable study and this study targeted a specific group of experts in our country.

### Conclusion

In the conventional laparoscopic surgical system, surgeons may value the proficiency of assistants, and most of them may feel uncomfortable with the unintended or not intuitive movement of laparoscopic cameras and devices. For the development of an AI-based surgical assistant system in the future, safe operation may be expected through lens cleaning, intuitive manipulation, and tissue damage minimization. Furthermore, an AI-based surgical assistant system is expected to replace approximately 41%-60% of the workforce, which may increase surgeons’ willingness to purchase such a system for reducing human resources and performing a comfortable, safe, and skilled operation. Conclusively, an AI-based surgical assistant system for AISS will become essential to enhance surgeons’ convenience, but it will be necessary to increase the safety and quality of surgery for patients.

## Abbreviations

AI: artificial intelligence
AISS: artificial intelligence–based solo surgery
